# Mode of Action of the Sesquiterpene Lactones Psilostachyin and Psilostachyin C on *Trypanosoma cruzi*

**DOI:** 10.1371/journal.pone.0150526

**Published:** 2016-03-03

**Authors:** Valeria P. Sülsen, Vanesa Puente, Daniela Papademetrio, Alcira Batlle, Virginia S. Martino, Fernanda M. Frank, María E. Lombardo

**Affiliations:** 1 Cátedra de Farmacognosia, Instituto de Química y Metabolismo del Fármaco (Universidad de Buenos Aires - Consejo Nacional de Investigaciones Científicas y Técnicas), Facultad de Farmacia y Bioquímica, Universidad de Buenos Aires, Buenos Aires, Argentina; 2 Centro de Investigaciones sobre Porfirinas y Porfirias (Universidad de Buenos Aires - Consejo Nacional de Investigaciones Científicas y Técnicas), Hospital de Clínicas José de San Martín, Universidad de Buenos Aires, Buenos Aires, Argentina; 3 Cátedra de Inmunología, Facultad de Farmacia y Bioquímica, Universidad de Buenos Aires, Buenos Aires, Argentina; 4 Instituto de Microbiología y Parasitología Médica (Universidad de Buenos Aires - Consejo Nacional de Investigaciones Científicas y Técnicas), Facultad de Medicina, UBA, Buenos Aires, Argentina; 5 Departamento de Química Biológica, Facultad de Ciencias Exactas y Naturales, Universidad de Buenos Aires, Buenos Aires, Argentina; Universidad de Chile, CHILE

## Abstract

*Trypanosoma cruzi* is the causative agent of Chagas’ disease, which is a major endemic disease in Latin America and is recognized by the WHO as one of the 17 neglected tropical diseases in the world. Psilostachyin and psilostachyin C, two sesquiterpene lactones isolated from *Ambrosia* spp., have been demonstrated to have trypanocidal activity. Considering both the potential therapeutic targets present in the parasite, and the several mechanisms of action proposed for sesquiterpene lactones, the aim of this work was to characterize the mode of action of psilostachyin and psilostachyin C on *Trypanosoma cruzi* and to identify the possible targets for these molecules. Psilostachyin and psilostachyin C were isolated from *Ambrosia tenuifolia* and *Ambrosia scabra*, respectively. Interaction of sesquiterpene lactones with hemin, the induction of oxidative stress, the inhibition of cruzipain and trypanothione reductase and their ability to inhibit sterol biosynthesis were evaluated. The induction of cell death by apoptosis was also evaluated by analyzing phosphatidylserine exposure detected using annexin-V/propidium iodide, decreased mitochondrial membrane potential, assessed with Rhodamine 123 and nuclear DNA fragmentation evaluated by the TUNEL assay. Both STLs were capable of interacting with hemin. Psilostachyin increased about 5 times the generation of reactive oxygen species in *Trypanosoma cruzi* after a 4h treatment, unlike psilostachyin C which induced an increase in reactive oxygen species levels of only 1.5 times. Only psilostachyin C was able to inhibit the biosynthesis of ergosterol, causing an accumulation of squalene. Both sesquiterpene lactones induced parasite death by apoptosis. Upon evaluating the combination of both compounds, and additive trypanocidal effect was observed. Despite their structural similarity, both sesquiterpene lactones exerted their anti-*T*. *cruzi* activity through interaction with different targets. Psilostachyin accomplished its antiparasitic effect by interacting with hemin, while psilostachyin C interfered with sterol synthesis.

## Introduction

American Trypanosomiasis or Chagas’ disease is caused by the protozoan parasite *Trypanosoma cruzi*. This parasitosis is endemic in 21 countries of Latin America and about 7 to 8 million people are affected worldwide. The parasite is transmitted to humans mainly by the faeces of triatomine bugs known as “kissing bugs”, by blood transfusion, organ transplantation, vertically and, to a lesser extent, by food contaminated with *T*. c*ruzi*. The disease has two clinical stages: the acute stage, in which 5% of children die, and a chronic stage. In the chronic phase, up to 30% of patients suffer from cardiac disorders and up to 10% suffer from digestive, neurological or mixed alterations. The outcome of this phase may sometimes be related to sudden death or heart failure due to progressive destruction of the heart muscle [1,2]. Current available drugs to treat this parasitosis, benznidazole and nifurtimox, have side effects that can lead to therapy discontinuation.

In recent years, an important progress in the knowledge of the biology and biochemistry of *T*. *cruzi* has been accomplished. These efforts have led to the identification of potential targets for Chagas’ disease chemotherapy. The ergosterol biosynthesis and trypanothione pathways, cysteine protease (cruzipain, CP) and thiol-dependent redox metabolism are considered the most promising biochemical targets for rational drug design [3].

Natural products have played an important role in the drug discovery process [4]. Regarding the treatment of parasitic diseases, the sesquiterpene lactone (STL) artemisinin and the alkaloid quinine, and their derivatives, are currently being used for the treatment of malaria [5]. STLs are an important group of natural compounds with pharmaceutical potential [6]. These compounds are mainly found in species of the Asteraceae family and have shown significant activity against trypanosomatids such as *Trypanosoma* spp. and *Leishmania* spp. [7–9].

**Fig 1:**
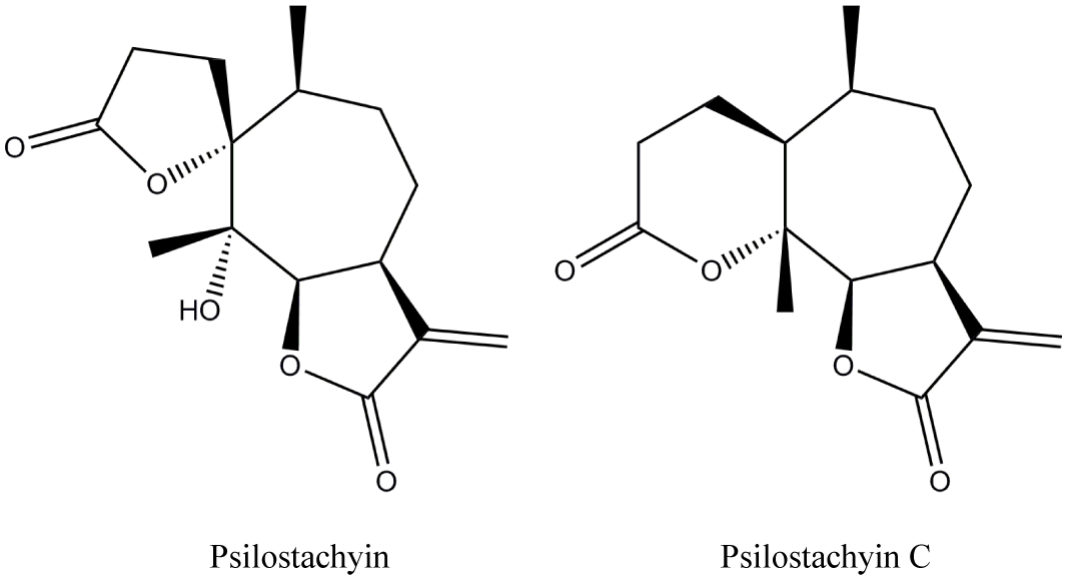
Chemical structure of psilostachyin and psilostachyin C isolated from *Ambrosia tenuifolia* and *Ambrosia scabra*, respectively.

In previous reports we have described the isolation of two sesquiterpene lactones (STLs) (Fig 1), psilostachyin (**Psi**) and psilostachyin C (**PsiC**) from species of the genus *Ambrosia* (Asteraceae) and we have described their antiprotozoal activity [10–14]. In this work we have evaluated the effects of **Psi** and **PsiC** on different targets and metabolic pathways of *T*. *cruzi*. The assays were selected taking in account some of the targets for the development of new trypanocidal drugs and some of the antiprotozoal mechanisms of action proposed for STLs, such as hemin interaction, ergosterol biosynthesis, generation of oxidative stress and apoptosis induction.

## Materials and Methods

### Test compounds and reagents

**Psi** and **PsiC** have been isolated from *Ambrosia tenuifolia* and *Ambrosia scabra*, respectively [5, 8]. The purity of **Psi** and **PsiC** was 96.8 and 95.5%, respectively as confirmed by high-performance liquid chromatography (HPLC) analysis.

Standard solutions of these compounds were prepared in dimethyl sulphoxide (DMSO) at a final concentration that never exceeded 0.5%. Hemin, artemisinin, NADPH, Rh123 and H_2_DCFDA were obtained from Sigma Chem. Co. (Saint Louis, MO, USA). Yeast extract, tryptose, powered beef liver and brain heart infusion were from Difco Laboratories (Sparks, MD, USA). Benznidazole (Bnz) was kindly provided by Roche (Argentina). All other chemicals were of the highest purity commercially available.

### Parasites

*Trypanosoma cruzi* epimastigotes (RA strain) were grown in a liquid medium containing 0.3% yeast extract, 0.9% tryptose, 0.4% dextrose, 1% disodium phosphate 2-hydrate, 0.36% sodium chloride, 0.04% potassium chloride, 0.15% powered beef liver, 0.5% brain heart infusion and 1.0 mg/100 mL hemin. Cultures were routinely maintained in the exponential phase by weekly passages at 28°C.

### *In vitro* assays for anti-*Trypanosoma cruzi* activity

To evaluate growth inhibition of *T*. *cruzi* epimastigotes, the percentage of inhibition (I%) and IC_50_ values (50% inhibitory concentration) for **Psi** and **PsiC** were estimated by counting the parasites using a Neubauer chamber, as previously described [11].

The variations in IC_50_ values of both STLs were analyzed for parasites cultured in the presence of different hemin concentration and to evaluate potential interaction between **Psi** and **PsiC**.

### Hemin binding assay

The interaction of the STLs with hemin was measured spectrophotometrically under reducing and non-reducing conditions by monitoring the Soret absorption band of hemin, using the methodology described by Taylor et. al (2004) with slight modifications [15]. The assay system under non-reducing conditions consisted of 0.23 M sodium phosphate buffer pH 7.4, 1% SDS, 7.5 μM hemin (protoporphyrin IX-Fe (III)) and different concentrations of **Psi** and **PsiC** (7.5 to 45 μM). Sodium dithionite (14 mM) was added to the solution to evaluate the interaction between the compounds and heme (protoporphyrin IX-Fe (II)). The absorption spectra were recorded using a Hewlett Packard 8452 –Diode Array spectrophotometer. The absorbance ratio at 430 and 400 nm (A_430_/A_400_) was used to quantify changes in the shape of the Soret band. Artemisinin was used as positive control.

### Cruzipain inhibition assay

A partially purified fraction containing cruzipain (CP) was obtained from a cell-free extract of *T*. *cruzi* epimastigotes by ConA-Sepharose affinity chromatography, as previously described [16]. The enzymatic activity was assayed with the synthetic chromogenic substrate Bz-Pro-Phe-Arg-pNA [17]. The reaction was monitored spectrophotometrically at 410 nm. The E-64 protease inhibitor was used as reference drug.

### Trypanothione reductase inhibition assay

A partially purified fraction containing trypanothione reductase (TryR) was obtained from a cell-free extract of *T*. *cruzi* epimastigotes as previously described [18] The enzymatic activity was determined following NADPH oxidation at 340 nm, at 25°C [19]. The corresponding non-enzymatic conversion controls were performed.

### Intracellular oxidative activity assay

The induction of intracellular oxidative stress was assessed using the oxidant-sensitive fluorescent probe H_2_DCFDA. *T*. *cruzi* epimastigotes growing in logarithmic phase were incubated with **Psi** or **PsiC** (35 μM) during 4, 8 or 24 h. Treated-parasites were harvested and stained for 45 min in the dark with 10 μM H_2_DCFDA at 37°C. As positive control, parasites were treated with 0.2 mM H_2_O_2_. The fluorescence intensity of dichlorofluorescein (DCF) in cells was then analyzed in a Becton Dickinson FACScalibur flow cytometer with an excitation wavelength of 480 nm and an emission wavelength of 530 nm. Results were expressed by the ratio Gm_t_/Gm_c_, where Gm_t_ and Gm_c_ correspond to the geometric mean of histograms obtained for treated and untreated (control) cells respectively [20].

### Electrochemical behaviour

Cyclic voltammograms for **Psi** and **PsiC** dissolved in 1% methanol were carried out using an EQMAT instrument with an EQSOFT Processor at a sweep rate of 0.2 V/s under a nitrogen atmosphere at room temperature and employing lithium perchlorate as supporting electrolyte. A three-electrode cell was used: a working electrode equipped with vitreous carbon; a gold wire as auxiliary electrode and a saturated calomel reference electrode [21].

### Analysis of ergosterol biosynthesis

*Trypanosoma cruzi* epimastigotes previously treated with 35 μM **Psi** or **PsiC** or 50 μM terbinafine (positive control) for 24 h were harvested by centrifugation at 10000 xg for 10 min and then washed once with 0.05 M sodium phosphate buffer pH 7.4. Cells were resuspended in 2.0 mL chloroform:methanol (2:1, v/v). Lipid extraction was complete after the suspension was sonicated in a Soniprep 150, MSE Ultrasonic Power employing two cycles of 30 s each and heated at 50°C during 30 min. After centrifugation at 500xg for 5 min, the organic phase was separated and the extraction was repeated twice with 1 mL of chloroform:methanol (2:1, v/v). The organic phases were then pooled, washed with 0.25 volume of 0.88% KCl and evaporated. Residues were dissolved in chloroform and analyzed by TLC employing silica-gel 60 plates (Merck) and developed in two runs, employing firstly hexane (to separate squalene of ergosterol) and then hexane:EtOAc (8:2, v/v) as eluents. Chromatograms were obtained by staining the plates with 1% CuSO_4_ in 8% H_3_PO_4_ and heating at 100°C. Ergosterol, lanosterol and squalene standards were run in parallel. Relative band intensities were determined by densitometry using the Scion Image software (Scion). Results were expressed in arbitrary units [17].

### Assays to evaluate cell death

These assays were developed with *T*. *cruzi* epimastigotes (2.5 x 10^7^ cells/mL) treated with **Psi** or **PsiC** (35 and 350 μM) during 8–72 h. Treated cells were harvested, washed and the following assays were carried out:

For the evaluation of parasite death, cell viability and phosphatidylserine (PS) exposure were measured. Annexin V-fluorescein isothiocyanate (FITC) (Invitrogen^™^) and propidium iodide (PI) staining were performed following the manufacturer’s instructions. As positive control, epimastigotes exposed to 30% fresh human serum for 2 h at 28°C were used. Parasite death was assessed by flow cytometry, acquiring 20,000 events per sample [21].

The mitochondrial membrane potential was assessed by Rh123 staining. After treatment with **Psi** and **PsiC**, parasites were suspended in PBS (2 x 10^6^ cells/mL) with 10 mg/L Rh123 and incubated for 15 min at 37°C. Trifluoromethoxy carbonyl cyanide phenyl hydrazone (FCCP) (250 nM) was used as positive control. Samples were analysed by flow cytometry with an excitation wavelength of 480 nm and an emission wavelength of 530 nm. A total of 20,000 events were acquired and the variations in the fluorescence were quantified using an index of variation (IV) calculated as IV = (Gm_t_−Gm_c_)/Gm_c_, where Gm_t_ and Gm_c_ corresponded to the geometric mean of histograms obtained for treated and untreated (control) cells respectively. Negative IV values corresponded to depolarization of the mitochondrial membrane. [22].

To analyze DNA fragmentation, a terminal deoxynucleotidyltransferase-mediated fluorescein dUTP nick end-labeling technique (DeadEnd Fluorometric TUNEL System, Promega, Madison, USA) was carried out following the manufacturer’s instructions. Parasites were pre-treated for 10 min at room temperature with 10 IU/mL DNase I prior to the TUNEL for positive control. A negative control was performed in the absence of the terminal transferase. Samples were incubated with 1 μg/mL 4,6-diamidino-2-phenylindole (DAPI) for DNA labeling, which allows visualization of the parasites’ nuclei. Samples were mounted in triplicate and examined immediately using an Olympus microscope [23].

### Drug interaction experiments

To evaluate the combinatory effect of **Psi** and **PsiC**, the IC_50_s for each of them, in the presence of different concentrations of the other, were calculated. The fractional inhibitory concentrations (FICs) were calculated as the ratio of the IC_50_ of one compound in combination and the IC_50_ of the compound alone. The FIC index (FICI) for the two compounds was the FIC of **Psi** plus the FIC of **Psi C**. FICI values ≤0.5 were considered as synergy, values >4.0 as antagonism and values in between 0.5 and 4 as no interaction [24].

### Statistical analysis

Results are representative of three to four separate experiments, performed in duplicate or triplicate. Data are expressed as means ± standard errors of the mean (SEMs). To calculate the IC_50_ values, I% values were plotted against the log of drug concentration (μM) and fitted to a sigmoidal curve determined by a non-linear regression (Sigma Plot 12 software). The significance of differences was evaluated using Student′s *t* test, or One-way ANOVA; p values < 0.05 (*) and < 0.01 (**) were considered significant. Flow cytometry data were analysed employing WinMDI 2.9 software.

## Results and Discussion

### Hemin binding

**Psi** and **PsiC** belong to the same phytochemical group as artemisinin, a STL currently used as an antimalarial drug. Since artemisinin has been demonstrated to exert its antiprotozoal activity through intracellular heme binding [9], it was of great interest to investigate the interaction between these STLs and hemin.

It is known that in the metabolism of heme-deficient parasites, hemin [protoporphyrin IX-Fe(III)] is a product of the digestion of hemoglobin. Hemin is then converted to hemozoin, a polymerized non-toxic form of heme [19]. To evaluate whether heme could be a target of **Psi** and **PsiC**, the affinity of both STLs to hemin was determined (Table 1). These STLs showed a considerable interaction with hemin, even higher than that shown by artemisinin. In fact, higher hemin:artemisinin ratios were required to achieve similar Soret band shifts to those exerted by Psi and PsiC. Under non-reducing conditions, the affinity of **PsiC** for hemin was higher than that of **Psi**. Under reducing conditions, **PsiC** decreased its affinity for hemin, unlike **Psi**, whose affinity for hemin was found to be slightly increased.

**Table 1:** Interaction of Psi and PsiC with hemin.

Drug (μM)	Molar ratio Hemin:Drug	Absorbance ratio A_430_/A_400_	
		(A)	(B)
Control	1:0	0.23	0.23
Artemisinin			
39.75	1:5	0.25	0.27*
79.50	1:10	0.28*	0.35*
119.25	1:15	0.32*	0.41*
**PsiC**			
7,65	1:1	0.27*	0.25
15.30	1:2	0.35*	0.27*
22.95	1:3	0.41*	0.30*
30.60	1:4	0.45*	0.34*
38.25	1:5	0.48*	0.35*
**Psi**			
7.65	1:1	0.26*	0.25
15.30	1:2	0.26*	0.27*
22.95	1:3	0.27*	0.28*
30.60	1:4	0.27*	0.30*
38.25	1:5	0.29*	0.32*
45.90	1:6	0.32*	0.37*

Hemin interaction was assayed spectrophotometrically under non-reducing (A) and reducing conditions (B), by monitoring the Soret band absorption.

*Significant differences (p<0.05) when compared to the control, as assessed by Student's *t*-test.

### *Trypanosoma cruzi* growth inhibition under different hemin concentrations

**Fig 2:**
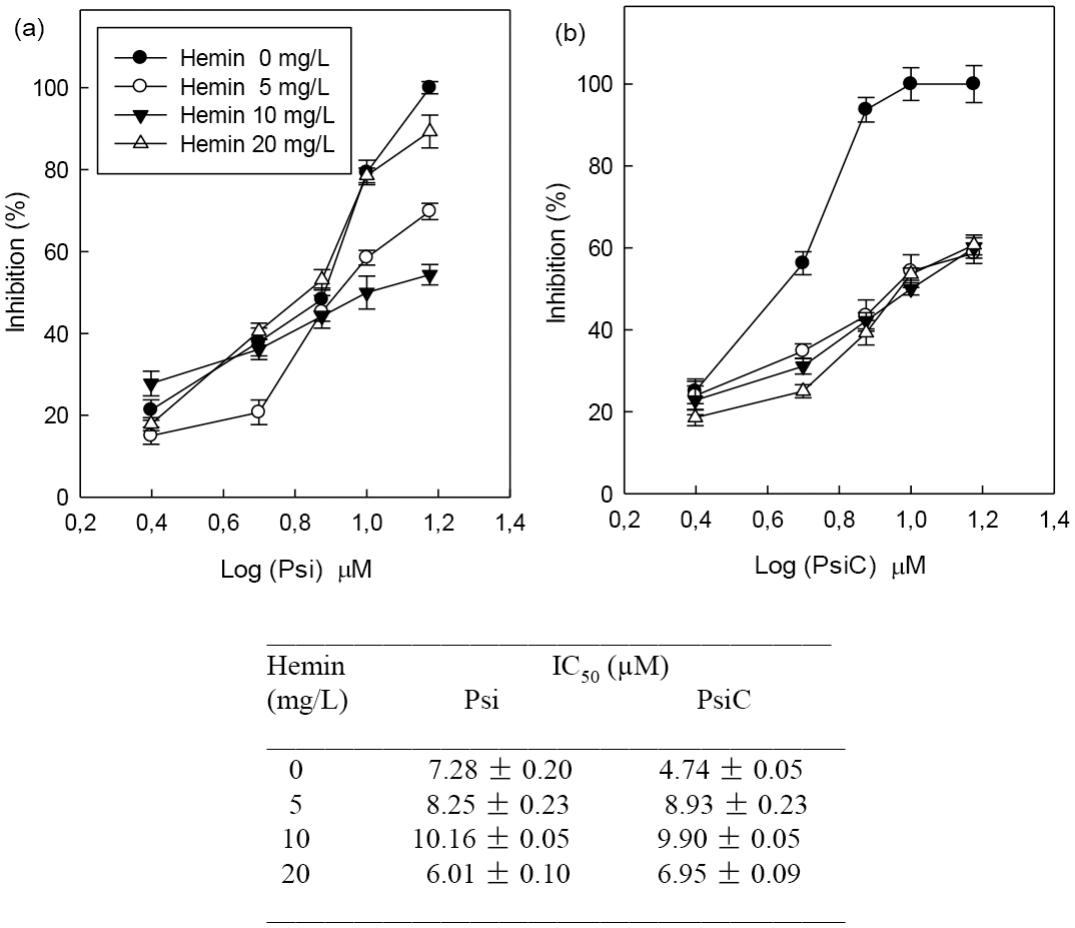
Effect of Psi and PsiC on epimastigotes cultured with different hemin concentrations. *T*. *cruzi* epimastigotes were grown at 28°C for 4 days with Psi (a) and PsiC (b) (2.5–15 μM) in culture medium containing different hemin concentrations (0, 5, 10 and 20 mg/L). (c) The IC_50_ (50% inhibitory concentration) values for each compound with different hemin concentrations were estimated by lineal regression analysis from the inhibition percentage values and the decimal logarithm (log) of drug concentration.

Taking into account that hemin is essential for parasite survival and considering its interaction with **Psi** and **PsiC**, the IC_50_ values for these STLs were calculated in the presence of different hemin concentrations (0–20 mg/L) (Fig 2). For the hemin concentration yielding optimum growth (5 mg/L), the antiparasitic effect shown by both STLs was similar. In the absence of hemin added to the medium, **PsiC** showed the highest inhibitory activity, whereas high levels of hemin were required to obtain the lowest IC_50_ for **Psi**. It has been reported that hemin itself (20 mg/L) has an inhibitory effect on *T*. *cruzi* epimastigotes growth [18]. We have observed such effect since 20 mg/L hemin decreased about 40% the optimal growth obtained with 5 mg/L hemin. When 10 μM **Psi** or **PsiC** were added to the culture medium, containing either 5 or 20 mg/L, inhibitory effects over 40% were observed, reaching values of 87% and 72% respectively (data not shown).

Our results showed that **Psi** and **PsiC** can bind to both protoporphyirin IX-Fe(III) (hemin) and protoporphyrin IX-Fe(II) (heme). The inhibition of hemin detoxification could lead to an oxidative stress within the parasite. **PsiC** showed higher affinity for hemin than for **Psi**, which was evidenced by the affinity test performed without parasites. The highest affinity of **PsiC** for hemin would justify the IC_50_ values obtained for 0 and 20 mg/L hemin. The absence of hemin in the medium increased the availability of the **PsiC** inside the parasite where the high affinity for intracellular hemin would allow this compound to manifest its maximum inhibitory capacity (IC_50_ 4.74 μM). In contrast, **Psi** showed a significant reduction in its IC_50_ value in the presence of 20 mg/L of hemin. This fact would indicate that the presence of high levels of hemin in the medium is a requirement to exert its maximum inhibitory activity.

### Oxidative stress induction

Taking into consideration that these compounds could exert their antiparasitic effect by inhibiting hemin detoxification, the generation of an oxidative stress should manifest inside the parasite. To test this hypothesis, concentrations and times of treatment were selected in order to prevent parasite death or to prevent response revertion.

**Fig 3:**
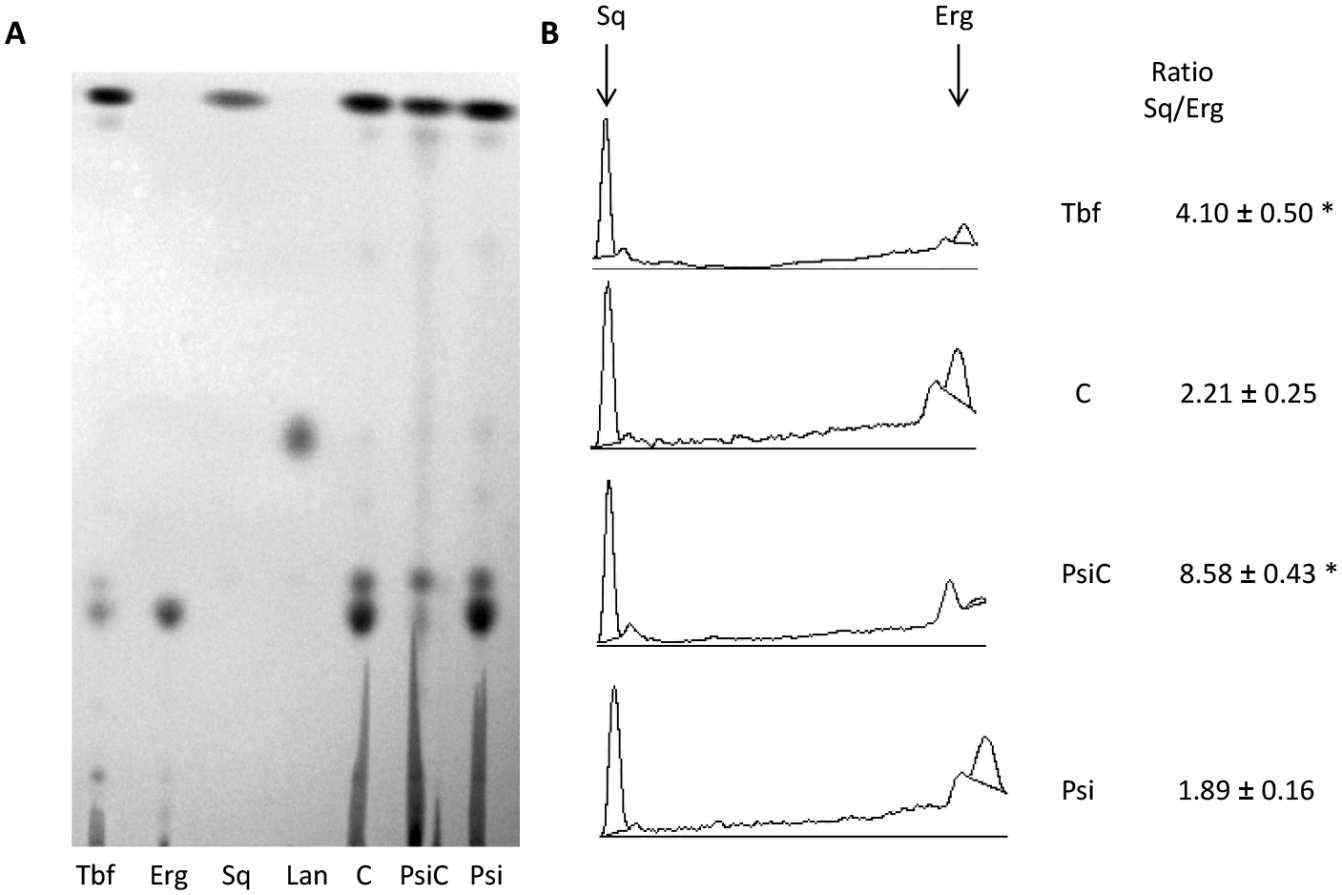
Intracellular oxidative stress during the treatment with Psi or PsiC. *T*. *cruzi* epimastigotes from a 4-day culture were incubated with 35 μM Psi or PsiC during 4, 8 or 24 h. Intracellular oxidative stress was evaluated by flow cytometry as indicated. (a) Histograms correspond to untreated cells (curve 1, control) and treated with 35 μM Psi for 4, 8 and 24 h (curves 2, 3 and 4, respectively). As positive control, parasites were treated with 0.2 mM H_2_O_2_ (curve 5). (b) Time course of the Gm_t_/Gm_c_ ratio for parasites treated with Psi or PsiC. p values < 0.05 (*) and < 0.01 (**) were considered significant.

For the evaluation of the oxidative stress induced by **Psi** and **PsiC**, *T*. *cruzi* epimastigotes cultured in the presence of **Psi** or **PsiC** (35 μM) during 4, 8 and 24 h were used. Results obtained by flow cytometry are shown in Fig 3.

The treatment with **Psi** induced a ~4.5-fold increase in the fluorescence of H_2_DCFDA-loaded epimastigotes after 4 h, and these values were sustained (more than 7-fold) until the end of the experiment (24 h). Markedly lower values were observed in parasites after 4 and 8 h of treatment with **PsiC**. These results are in accordance with our previous findings obtained in experiments performed on *Leishmania* spp. in which **Psi**, but not **PsiC**, in short-time treatment, increased the generation of ROS within the parasites [10]. At 24 h, both STLs showed a similar increase of around 7-fold in cell fluorescence intensity.

When evaluating the electrochemical behavior of **Psi** and **PsiC**, neither cathodic nor anodic peaks, corresponding to reactions of reduction and oxidation respectively, were observed at the tested concentrations and potentials (data not shown). The absence of cathodic and anodic peaks was observed even in the presence of glutathione (GSH), added at the ratio GSH: STL (1:1) and (2:1) (data not shown). These results allow inferring that the modification of the intracellular oxidative state of the parasites could not be attributed to the reduction of **Psi** and **PsiC**.

### CP and TryR inhibition

CP and TryR enzymes are present only in trypanosomatids and absent in mammalian cells. CP is a cysteine protease, relevant for the parasite metabolism, which is considered an important candidate for the development of new trypanocidal drugs [25].

TryR is an oxidoreductase, also specific for trypanosomatids, but parasites can survive with up to 10% of TRyR activity [26]. Because of this reason, not always a good correlation between the enzyme inhibition and the antiprotozoal efficiency has been observed, possibly due to a limited entrance of the drug to the parasite. Nevertheless, according to these authors, this enzyme can still be considered a potential target for drug design. A possible mechanism of action of STLs as trypanocidal agents could involve the trypanothione redox system, since STLs contain an α,β-unsaturated-γ-lactone moiety in its structure.

Taking in account these considerations, the effects of **Psi** and **PsiC** were evaluated on CP and TryR. Neither **Psi** nor **PsiC** produced inhibition of these enzymes at the assayed doses (10, 20 and 50 μM) (data not shown). Our results are in accordance with those reported by other authors [27], demonstrating that TryR would not be a target of this class of compounds.

### Sterol biosynthesis inhibition

**Fig 4:**
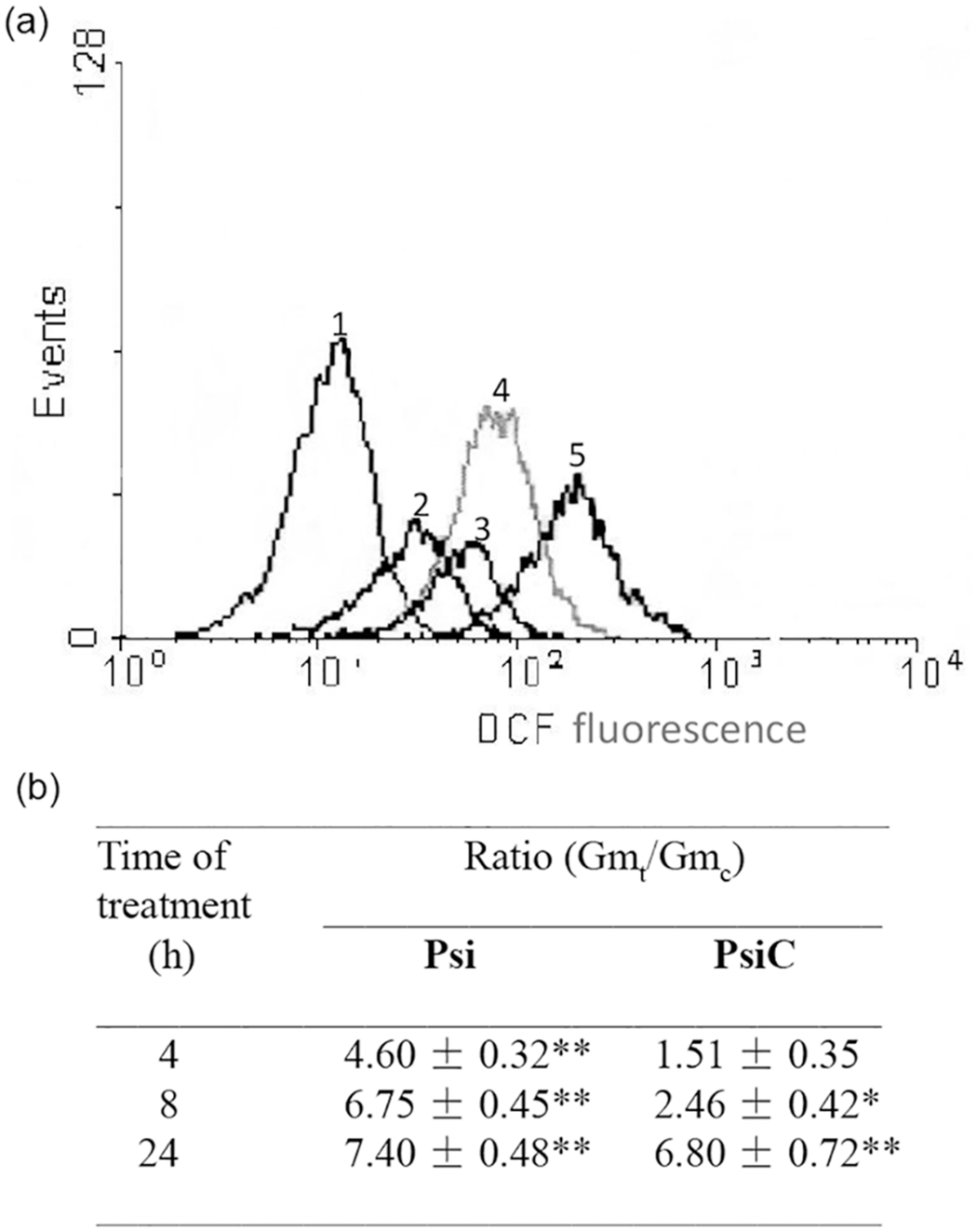
Effect of Psi and PsiC on sterol biosynthesis. A) TLC analysis of lipid extracts from *T*. *cruzi* epimastigotes treated with Psi and PsiC. Tbf: terbinafine (positive control), Erg: ergosterol, Sq: squalene, Lan: lanosterol, C: extract without treatment. B) Relative intensities of bands were quantified by densitometry using Scion Image software (Scion). Experiments were performed in triplicate. Results are expressed in arbitrary units. p values < 0.05 (*) were considered as significant.

The biosynthesis of ergosterol has proved to be crucial for the growth of *T*. *cruzi*. Lipids were extracted from epimastigotes and analysed by TLC after 24 h incubation in the presence or absence of **Psi** or **PsiC** (35 μM) (Fig 4). No accumulation of lanosterol (Lan) was observed for any treatment with the compounds. A decrease of ergosterol (Erg) levels was observed in **PsiC**-treated parasites. A ratio squalene (Sq)/Erg of 8.58±0.43, 4-fold higher than that obtained in the untreated control (C), was found for these parasites, while terbinafine-treated parasites (50 μM, positive control) presented a Sq/Erg ratio of 4.10±0.50. The different lipid profiles obtained with parasites treated either with **Psi** or with **PsiC** suggests that the biosynthesis of Erg could be a target of **PsiC**, probably due to an inhibition of the Sq epoxidase activity.

### Apoptosis induction

STLs have proved to induce programmed cell death in trypanosomatids [10]. Since apoptosis has been pointed as one of the mechanisms by which STLs exert their antiprotozoal activity, we have evaluated the effect of **Psi** and **PsiC** on the induction of apoptosis on *T*. *cruzi*.

**Fig 5:**
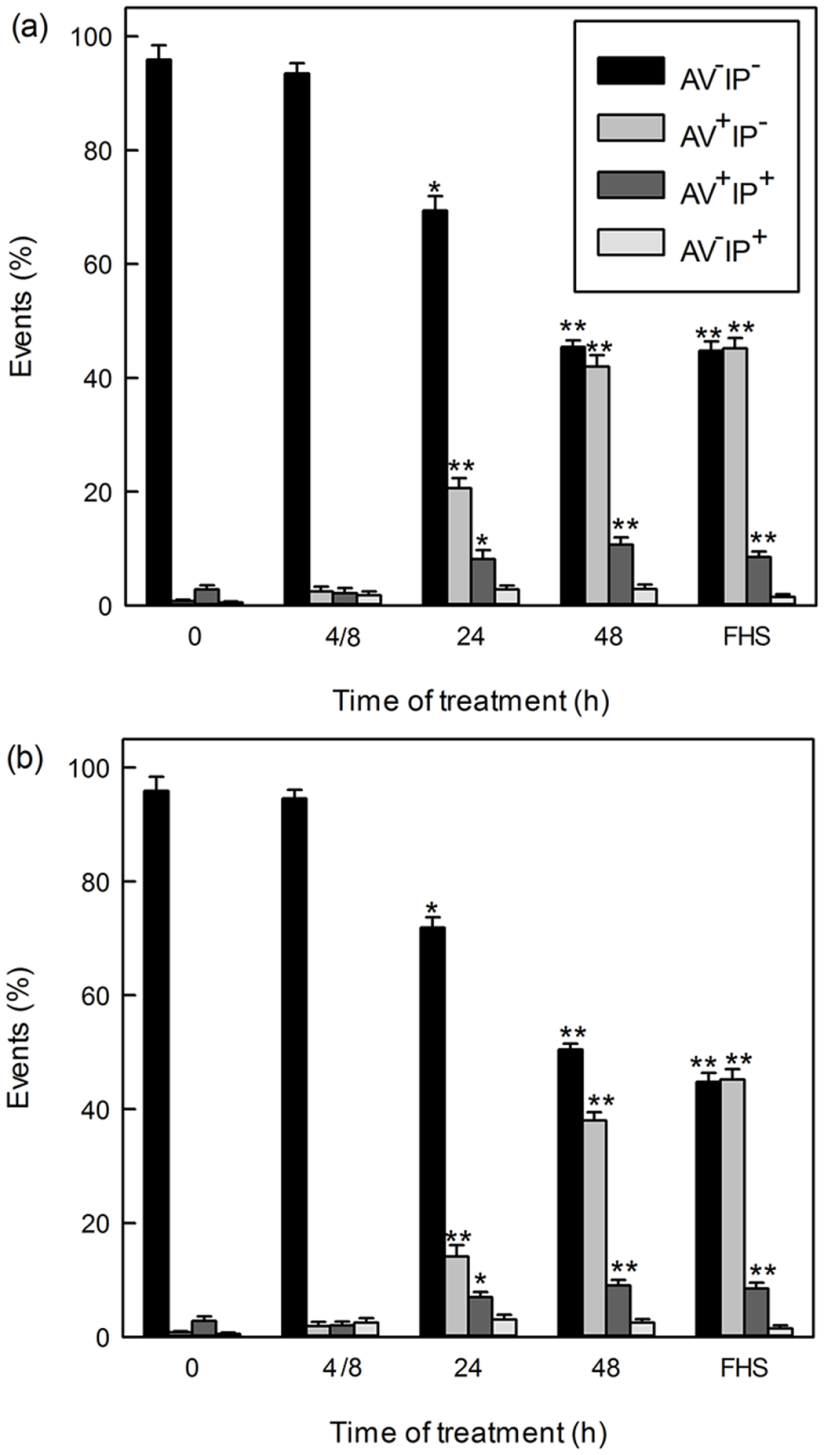
Effect of Psi and PsiC on cell death. Figs a-c: *T*. *cruzi* epimastigotes were treated with Psi and PsiC 35 μM during 8, 24 and 48 h and stained with Annexin V-FITC/PI (a and b). Bars correspond to AV^-^PI^-^: viable cells, AV^+^PI^-^: early apoptotic cells, AV^+^PI^+^: late apoptotic cells and AV^-^PI^+^: necrotic cells. Epimastigotes exposed to 30% fresh human serum (FHS) for 2 h at 28°C were used as positive control. p values < 0.05 (*) and < 0.01 (**) were considered significant.

To evaluate cell death and mitochondrial damage, parasites treated with **Psi** or **PsiC** at 35 μM for 8, 24 and 48 h were used. Annexin-V FITC/PI staining was used as a parameter to detect apoptotic cells. Results demonstrated that the number of apoptotic cells increased during the treatment with both STLs in a time-dependent manner (Fig 5a and 5b). Results were similar for both compounds. The most significant differences in the level of apoptotic cells were observed for early apoptotic cells for which values of 0.8%, 2.5%, 20.6% and 42.0% (**Psi)** or 0.8%, 1.9%, 14.1% and 38% (**PsiC)** were obtained for 0, 8, 24 and 48 h of treatment. The number of late apoptotic cells also increased with time of treatment but in a less marked and significant manner, reaching values of 10.7% (**Psi**) and 9.0% (**PsiC**) at 48 h of treatment. The number of viable non-apoptotic cells after 48 h of treatment reached values of 45.4% and 50.5% for **Psi** and **PsiC**, respectively *vs* 95.9% for untreated control (0 h).

The mitochondrial membrane depolarization was evident at 24 h of treatment with IV values of -0.45 (**Psi**) and -0.72 (**PsiC**), increasing by 51% (**Psi)** or remaining at similar levels (**PsiC**) up to 48h (Table 2). Depolarized cells reached values of 73% (**Psi**) and 85% (**PsiC**) of the evaluated cells after 48 h of treatment, *vs* 33% for the untreated control (data not shown).

**Table 2:** Flow cytometry analysis of *T*. *cruzi* epimastigotes treated with Psi or PsiC and labeled with Rh123.

Time of treatment (h)	Control	Psi		PsiC		FCCP	
	Gm	Gm	IV	Gm	IV	Gm	IV
4/8^a^	234.18	228.30	-0.03	225.48	-0.04	81.96**	-0.65
24	243.65	135.19**	-0.45	68.95**	-0.72	71.20**	-0.71
48	305.04	98.25**	-0.68	66.93**	-0.78	94.56**	-0.69

^a^For 4 and 8 h of treatment similar results were obtained.

Alterations in the fluorescence for Rh123 were expressed as index of variation (IV) calculated as IV = (Gm_t_—Gm_c_)/Gm_c_, where Gm_t_ and Gm_c_ corresponded to the geometric mean of histograms obtained for treated and untreated (control) cells. Trifluoremethoxy carbonyl cyanide phenyl hydrazone (FCCP) was used as positive control.

^(^**^)^ p<0.01 significance of differences between treated and control parasites.

**Fig 6:**
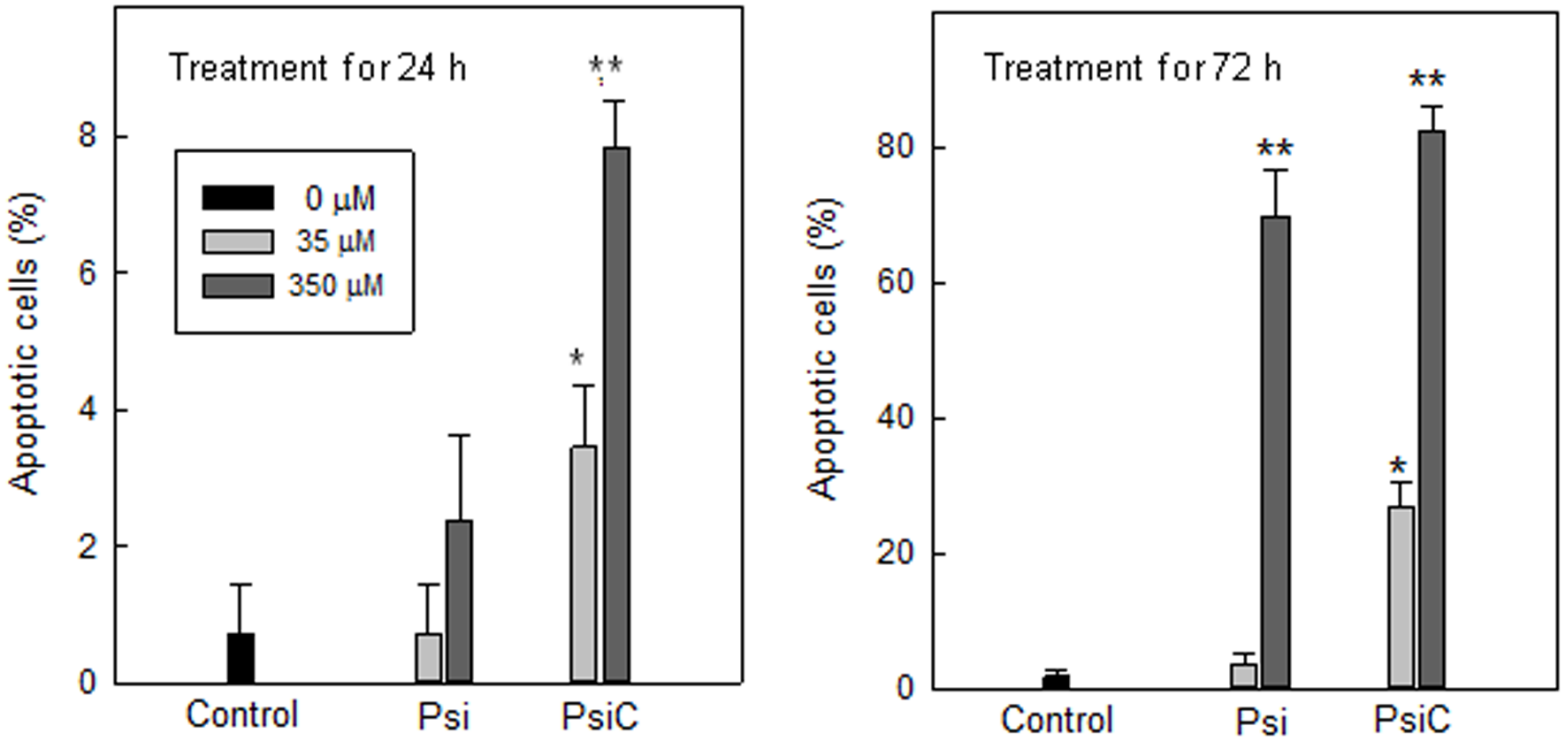
Effect of Psi and PsiC on DNA fragmentation. Nuclear fragmentation was analyzed by the TUNEL assay. Parasites were treated with Psi and PsiC 35 and 350 μM during 24 and 72 h. p values < 0.05 (*) and < 0.01 (**) were considered significant.

Finally, apoptotic cells were evaluated by the TUNEL assay. After 72 h, both STLs were able to induce nuclear fragmentation in 82.9% (**PsiC)** and 72.2% (**Psi**) of the parasites, when high concentrations (350 μM) were employed. **PsiC** was found to be a stronger apoptosis inductor, since smaller amounts (35μM) stimulated cell death in a 25.5% of the treated *T*. *cruzi* epimastigotes. Moreover, after 24 h treatment (350 μM), this compound induced a significant amount of apoptotic cells (7.9 ± 1.4%) (Fig 6).

Treatment with either **Psi** or **PsiC** induced time-dependent changes in the exposure of PS on the outer surface layer of the plasma membrane and in the mitochondrial membrane potential (early apoptotic events). Although DNA fragmentation could be seen by TUNEL staining for both compounds, **Psi** required higher concentrations and longer times of exposure than **PsiC**.

Considering that cell death by apoptosis was observed when high concentrations of STLs were used and after a long period of treatment, it is unlikely that the trypanocidal effect of **Psi** and **PsiC** could be mediated by programmed cell death induction.

### Drug interaction experiments

**Fig 7:**
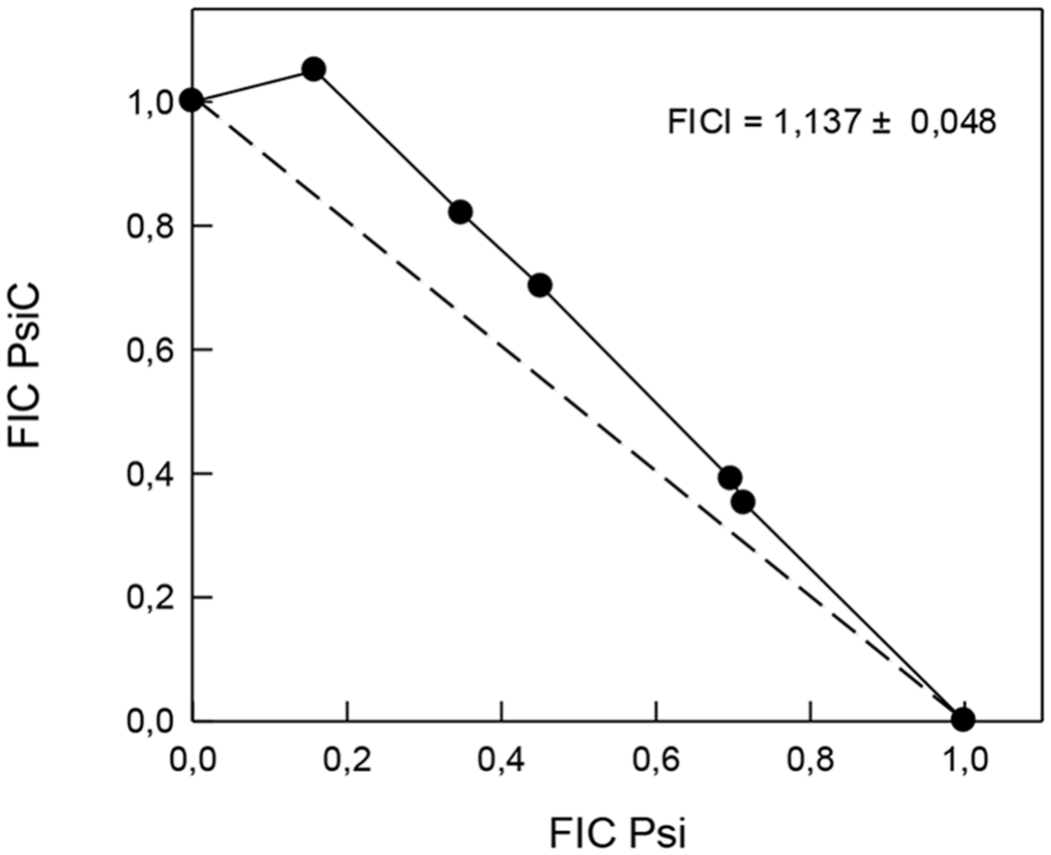
Isobologram describing the interaction between Psi and PsiC against *T*. *cruzi* epimastigotes. The fractional inhibitory concentrations (FICs) were calculated as the ratio between the IC_50_ of one compound in combination and the IC_50_ of the compound alone. The FIC index (FICI) for the two compounds was determined as the FIC of **Psi** plus the FIC of **Psi C**. FICI values between 0.5 and 4.0 indicate no interaction between **PsiC** and **Psi**.

Nowadays, the combination of drugs is a useful therapeutic approach to improve efficacy and to reduce side effects. To evaluate a possible interaction between **Psi** and **PsiC**, the combined effects of both STLs was investigated. The FICI was 1.11 ± 0.05 (Fig 7). The association of **Psi** and **PsiC** revealed an additive trypanocidal effect, which could be related to the interaction with different targets to exert their antiparasitic activity.

## Conclusions

In conclusion, this work provides a characterization of the mode of action of the STLs **Psi** and **PsiC** on *T*. *cruzi*. Although there are structural similarities between both STLs, our results have proved that they exert their anti-*T*. *cruzi* activity by acting on different targets. This work suggests that the interaction with heme seems to be one of the mechanism of action for **Psi**, while **PsiC** would act by inhibiting the synthesis of sterols. The combination of **Psi** and **PsiC** produced an additive effect, supporting the previous findings indicating the existence of different mechanisms of action of these compounds. This association may be further investigated as a potential new therapeutic modality for the treatment of Chagas' disease.

## Abbreviations

Bnz: Benznidazole
DCF: diclorofluorescein
FCCP: trifluoromethoxy carbonyl cyanide phenyl hydrazone
H_2_DCFDA: 2',7'-dichlorodihydrofluorescein diacetate
PI: propidium iodide
PS: phosphatidylserine
Rh123: rhodamine 123
ROS: reactive oxygen species
TCA: trichloroacetic acid
